# Supplementary material for: Burden of post-COVID-19 syndrome and implications for healthcare service planning: A population-based cohort study
Source: PLoS One. 2021 Jul 12;16(7):e0254523. doi: 10.1371/journal.pone.0254523 (PMC8274847; doi:10.1371/journal.pone.0254523)
Supplement: S1 Table — (DOCX) [file pone.0254523.s001.docx]

**S1 Table. Comparison of population characteristics of participants of the Zurich SARS-CoV-2 Cohort study and individuals not participating in the study.**

| **Variable** | **Participants included in analysis** *^a^*, N=431 | **Enrolled Participants** *^b,c^*, N=442 | **Nonparticipants** *^b^*, N=858 |
| --- | --- | --- | --- |
| **Age group (years)** |  |  |  |
| 18-39 | 164 (38.1%) | 170 (38.4%) | 325 (37.9%) |
| 40-64 | 205 (47.6%) | 208 (47.1%) | 352 (41.0%) |
| ≥65 | 62 (14.4%) | 64 (14.5%) | 181 (21.1%) |
| **Sex** |  |  |  |
| Female | 214 (49.7%) | 218 (49.3%) | 415 (48.4%) |
| Male | 217 (50.3%) | 224 (50.7%) | 440 (51.3%) |
| *Missing* | *0* | *0* | *3 (0.3%)* |
| **Initial symptoms** |  |  |  |
| Symptomatic | 385 (89.3%) | 370 (83.7%) | 692 (80.7%) |
| Asymptomatic | 46 (10.7%) | 8 (1.8%) | 36 (4.2%) |
| *Missing* | *0* | *64 (14.5%)* | *130 (15.1%)* |
| **Time from symptom onset to diagnosis (days)** |  |  |  |
| Median (IQR) | - *^d^* | 2 (1 to 5) | 2 (1 to 5) |
| **Hospitalization** | 81 (18.8%) | 58 (13.2%) | 205 (23.9%) |

*Legend: ^a^ Data presented refers to that collected in the Zurich SARS-CoV-2 Cohort study. ^b^ Data presented refers to that recorded in contact tracing files within the Department of Health. ^c^ Includes all participants who consented to participate in the Zurich SARS-CoV-2 Cohort study (i.e., including 6 individuals with suspected reinfection and 5 individuals that never filled a questionnaire). ^d^ Time from symptom onset to diagnosis was not retrospectively elicited in our study.*
