# Supplementary material for: Burden of post-COVID-19 syndrome and implications for healthcare service planning: A population-based cohort study
Source: PLoS One. 2021 Jul 12;16(7):e0254523. doi: 10.1371/journal.pone.0254523 (PMC8274847; doi:10.1371/journal.pone.0254523)
Supplement: S2 Table — (DOCX) [file pone.0254523.s002.docx]

**S2 Table. Results from univariable and multivariable logistic regression models for the outcome of not having fully recovered at six to eight months after diagnosis.**

| **Variable** |  | **Univariable** | | |  | **Multivariable** *^a^* | | |
| --- | --- | --- | --- | --- | --- | --- | --- | --- |
|  | **N** | **OR** | **95% CI** | **p-value** |  | **OR** | **95% CI** | **p-value** |
| **Age group (years)** | *385* |  |  |  |  |  |  |  |
| 18-39 |  | — | — |  |  | — | — |  |
| 40-64 |  | 1.96 | 1.20 to 3.25 | 0.008 |  | 1.59 | 0.93 to 2.73 | 0.093 |
| ≥65 |  | 1.45 | 0.70 to 2.95 | 0.31 |  | 0.97 | 0.41 to 2.20 | 0.94 |
| **Sex** | *385* |  |  |  |  |  |  |  |
| Male |  | — | — |  |  | — | — |  |
| Female |  | 1.7 | 1.09 to 2.67 | 0.02 |  | 1.89 | 1.18 to 3.07 | 0.009 |
| **Time since diagnosis (days)** | *385* | 1.00 | 0.99 to 1.00 | 0.69 |  | 0.99 | 0.99 to 1.00 | 0.10 |
| **Initial symptom severity** | *385* |  |  |  |  |  |  |  |
| Mild to moderate |  | — | — |  |  | — | — |  |
| Severe to very severe |  | 2.5 | 1.60 to 3.94 | <0.001 |  | 2.05 | 1.27 to 3.34 | 0.003 |
| **Initial hospitalization** | *385* |  |  |  |  |  |  |  |
| No |  | — | — |  |  | — | — |  |
| Yes |  | 1.75 | 1.03 to 2.96 | 0.038 |  | 1.17 | 0.63 to 2.16 | 0.61 |
| **Initial ICU stay** | *385* |  |  |  |  |  |  |  |
| No |  | — | — |  |  | — | — |  |
| Yes |  | 1.06 | 0.23 to 3.89 | 0.93 |  | 0.55 | 0.10 to 2.49 | 0.45 |
| **Smoking status** | *382* |  |  |  |  |  |  |  |
| Non-smoker |  | — | — |  |  | — | — |  |
| Ex-smoker |  | 1.44 | 0.88 to 2.36 | 0.15 |  | 1.48 | 0.87 to 2.52 | 0.14 |
| Smoker |  | 1.21 | 0.61 to 2.32 | 0.56 |  | 1.61 | 0.78 to 3.24 | 0.19 |
| **Body mass index** | *379* | 1.07 | 1.02 to 1.12 | 0.004 |  | 1.04 | 0.99 to 1.09 | 0.15 |
| **Comorbidities** | *384* |  |  |  |  |  |  |  |
| No |  | — | — |  |  | — | — |  |
| Yes |  | 2.21 | 1.40 to 3.48 | <0.001 |  | 2.08 | 1.24 to 3.50 | 0.005 |
| *Legend: OR = Odds Ratio, CI = Confidence Interval, ICU = Intensive Care Unit; ^a^ adjusted for age group, sex, initial hospitalization, symptom severity, and comorbidities.* | | | | | | | | |
