# Supplementary material for: Burden of post-COVID-19 syndrome and implications for healthcare service planning: A population-based cohort study
Source: PLoS One. 2021 Jul 12;16(7):e0254523. doi: 10.1371/journal.pone.0254523 (PMC8274847; doi:10.1371/journal.pone.0254523)
Supplement: S3 Table — (DOCX) [file pone.0254523.s003.docx]

**S3 Table**. **Results from univariable and multivariable logistic regression models for the outcome of fatigue at six to eight months after diagnosis.**

| **Variable** |  | **Univariable** | | |  | **Multivariable** *^a^* | | |
| --- | --- | --- | --- | --- | --- | --- | --- | --- |
|  | **N** | **OR** | **95% CI** | **p-value** |  | **OR** | **95% CI** | **p-value** |
| **Age group (years)** | *426* |  |  |  |  |  |  |  |
| 18-39 |  | — | — |  |  | — | — |  |
| 40-64 |  | 0.58 | 0.38 to 0.89 | 0.012 |  | 0.59 | 0.39 to 0.91 | 0.018 |
| ≥65 |  | 0.4 | 0.21 to 0.73 | 0.003 |  | 0.41 | 0.21 to 0.78 | 0.007 |
| **Sex** | *426* |  |  |  |  |  |  |  |
| Male |  | — | — |  |  | — | — |  |
| Female |  | 1.44 | 0.98 to 2.12 | 0.062 |  | 1.38 | 0.94 to 2.04 | 0.10 |
| **Time since diagnosis (days)** | *426* | 1.00 | 1.00 to 1.01 | 0.85 |  | 1.00 | 1.00 to 1.01 | 0.52 |
| **Initial symptom severity** | *426* |  |  |  |  |  |  |  |
| Asymptomatic |  | — | — |  |  | — | — |  |
| Mild to moderate |  | 0.99 | 0.52 to 1.86 | 0.96 |  | 0.96 | 0.50 to 1.83 | 0.89 |
| Severe to very severe |  | 1.34 | 0.69 to 2.59 | 0.38 |  | 1.38 | 0.69 to 2.73 | 0.36 |
| **Initial hospitalization** | *426* |  |  |  |  |  |  |  |
| No |  | — | — |  |  | — | — |  |
| Yes |  | 0.77 | 0.47 to 1.26 | 0.30 |  | 1.00 | 0.59 to 1.71 | 0.99 |
| **Initial ICU stay** | *426* |  |  |  |  |  |  |  |
| No |  | — | — |  |  | — | — |  |
| Yes |  | 2.96 | 0.71 to 20.0 | 0.18 |  | 4.63 | 1.02 to 32.9 | 0.07 |
| **Smoking status** | *424* |  |  |  |  |  |  |  |
| Non-smoker |  | — | — |  |  | — | — |  |
| Ex-smoker |  | 1.35 | 0.87 to 2.11 | 0.18 |  | 1.58 | 1.00 to 2.52 | 0.05 |
| Smoker |  | 1.47 | 0.83 to 2.63 | 0.19 |  | 1.27 | 0.71 to 2.31 | 0.42 |
| **Body mass index** | *419* | 1.01 | 0.97 to 1.05 | 0.52 |  | 1.04 | 1.00 to 1.09 | 0.08 |
| **Comorbidities** | *426* |  |  |  |  |  |  |  |
| No |  | — | — |  |  | — | — |  |
| Yes |  | 0.91 | 0.61 to 1.36 | 0.65 |  | 1.27 | 0.81 to 2.01 | 0.30 |
| *Legend: OR = Odds Ratio, CI = Confidence Interval, ICU = Intensive Care Unit; ^a^ adjusted for age group, sex, and initial hospitalization.* | | | | | | | | |
