# Supplementary material for: Burden of post-COVID-19 syndrome and implications for healthcare service planning: A population-based cohort study
Source: PLoS One. 2021 Jul 12;16(7):e0254523. doi: 10.1371/journal.pone.0254523 (PMC8274847; doi:10.1371/journal.pone.0254523)
Supplement: S4 Table — (DOCX) [file pone.0254523.s004.docx]

**S4 Table. Results from univariable and multivariable logistic regression models for the outcome of mMRC dyspnea grade ≥1 at six to eight months after diagnosis.**

| **Variable** |  | **Univariable** | | |  | **Multivariable** *^a^* | | |
| --- | --- | --- | --- | --- | --- | --- | --- | --- |
|  | **N** | **OR** | **95% CI** | **p-value** |  | **OR** | **95% CI** | **p-value** |
| **Age group (years)** | *395* |  |  |  |  |  |  |  |
| 18-39 |  | — | — |  |  | — | — |  |
| 40-64 |  | 1.61 | 0.96 to 2.76 | 0.077 |  | 0.79 | 0.41 to 1.49 | 0.46 |
| ≥65 |  | 2.88 | 1.45 to 5.73 | 0.002 |  | 0.90 | 0.37 to 2.16 | 0.82 |
| **Sex** | *395* |  |  |  |  |  |  |  |
| Male |  | — | — |  |  | — | — |  |
| Female |  | 1.68 | 1.06 to 2.70 | 0.029 |  | 2.24 | 1.31 to 3.87 | 0.003 |
| **Time since diagnosis (days)** | *395* | 1.00 | 0.99 to 1.01 | 0.74 |  | 1.00 | 0.99 to 1.01 | 0.92 |
| **Initial symptom severity** | *395* |  |  |  |  |  |  |  |
| Asymptomatic |  | — | — |  |  | — | — |  |
| Mild to moderate |  | 0.87 | 0.40 to 2.07 | 0.74 |  | 1.00 | 0.40 to 2.73 | >0.99 |
| Severe to very severe |  | 1.93 | 0.89 to 4.54 | 0.11 |  | 1.42 | 0.57 to 3.87 | 0.47 |
| **Initial hospitalization** | *395* |  |  |  |  |  |  |  |
| No |  | — | — |  |  | — | — |  |
| Yes |  | 4.06 | 2.39 to 6.91 | <0.001 |  | 4.17 | 2.23 to 7.91 | <0.001 |
| **Initial ICU stay** | *395* |  |  |  |  |  |  |  |
| No |  | — | — |  |  | — | — |  |
| Yes |  | 4.05 | 1.05 to 16.7 | 0.04 |  | 1.05 | 0.22 to 5.31 | 0.95 |
| **Smoking status** | *393* |  |  |  |  |  |  |  |
| Non-smoker |  | — | — |  |  | — | — |  |
| Ex-smoker |  | 1.62 | 0.97 to 2.69 | 0.06 |  | 1.66 | 0.92 to 3.00 | 0.093 |
| Smoker |  | 0.88 | 0.41 to 1.78 | 0.73 |  | 1.31 | 0.57 to 2.85 | 0.51 |
| **Body mass index** | *388* | 1.13 | 1.08 to 1.20 | <0.001 |  | 1.14 | 1.08 to 1.20 | <0.001 |
| **Respiratory condition** | *388* |  |  |  |  |  |  |  |
| No |  | — | — |  |  | — | — |  |
| Yes |  | 2.36 | 1.10 to 4.95 | 0.024 |  | 1.71 | 0.70 to 4.01 | 0.22 |
| **Comorbidities** | *395* |  |  |  |  |  |  |  |
| No |  | — | — |  |  | — | — |  |
| Yes |  | 3.93 | 2.44 to 6.40 | <0.001 |  | 2.71 | 1.38 to 5.36 | 0.004 |
| *Legend: OR = Odds Ratio, CI = Confidence Interval, ICU = Intensive Care Unit; ^a^ adjusted for age group, sex, initial hospitalization, smoking, respiratory comorbidity, and body mass index.* | | | | | | | | |
