# Supplementary material for: Burden of post-COVID-19 syndrome and implications for healthcare service planning: A population-based cohort study
Source: PLoS One. 2021 Jul 12;16(7):e0254523. doi: 10.1371/journal.pone.0254523 (PMC8274847; doi:10.1371/journal.pone.0254523)
Supplement: S5 Table — (DOCX) [file pone.0254523.s005.docx]

**S5 Table**. **Results from univariable and multivariable logistic regression models for the outcome of depression at six to eight months after diagnosis.**

| **Variable** |  | **Univariable** | | |  | **Multivariable** *^a^* | | |
| --- | --- | --- | --- | --- | --- | --- | --- | --- |
|  | **N** | **OR** | **95% CI** | **p-value** |  | **OR** | **95% CI** | **p-value** |
| **Age group (years)** | *428* |  |  |  |  |  |  |  |
| 18-39 |  | — | — |  |  | — | — |  |
| 40-64 |  | 1.05 | 0.66 to 1.69 | 0.83 |  | 0.97 | 0.59 to 1.59 | 0.91 |
| ≥65 |  | 1.19 | 0.60 to 2.28 | 0.61 |  | 1.18 | 0.56 to 2.42 | 0.65 |
| **Sex** | *428* |  |  |  |  |  |  |  |
| Male |  | — | — |  |  | — | — |  |
| Female |  | 0.70 | 0.45 to 1.08 | 0.11 |  | 0.74 | 0.47 to 1.15 | 0.18 |
| **Time since diagnosis (days)** | *428* | 1.00 | 0.99 to 1.00 | 0.65 |  | 1.00 | 0.99 to 1.00 | 0.28 |
| **Initial symptom severity** | *428* |  |  |  |  |  |  |  |
| Asymptomatic |  | — | — |  |  | — | — |  |
| Mild to moderate |  | 0.84 | 0.40 to 1.92 | 0.67 |  | 0.85 | 0.40 to 1.94 | 0.69 |
| Severe to very severe |  | 2.08 | 0.99 to 4.71 | 0.062 |  | 2.05 | 0.96 to 4.69 | 0.074 |
| **Initial hospitalization** | *428* |  |  |  |  |  |  |  |
| No |  | — | — |  |  | — | — |  |
| Yes |  | 1.42 | 0.82 to 2.39 | 0.20 |  | 1.05 | 0.57 to 1.89 | 0.88 |
| **Initial ICU stay** | *428* |  |  |  |  |  |  |  |
| No |  | — | — |  |  | — | — |  |
| Yes |  | 0.81 | 0.12 to 3.42 | 0.80 |  | 0.47 | 0.07 to 2.17 | 0.37 |
| **Smoking status** | *426* |  |  |  |  |  |  |  |
| Non-smoker |  | — | — |  |  | — | — |  |
| Ex-smoker |  | 1.18 | 0.72 to 1.94 | 0.51 |  | 1.21 | 0.72 to 2.01 | 0.48 |
| Smoker |  | 1.56 | 0.83 to 2.84 | 0.16 |  | 1.71 | 0.89 to 3.21 | 0.10 |
| **Body mass index** | *421* | 1.02 | 0.98 to 1.06 | 0.40 |  | 1.02 | 0.97 to 1.06 | 0.51 |
| **Comorbidities** | *428* |  |  |  |  |  |  |  |
| No |  | — | — |  |  | — | — |  |
| Yes |  | 1.48 | 0.94 to 2.31 | 0.086 |  | 1.41 | 0.84 to 2.34 | 0.19 |
| **Education** | *426* |  |  |  |  |  |  |  |
| None or mandatory school |  | — | — |  |  | — | — |  |
| Vocational training or specialized baccalaureate |  | 0.34 | 0.13 to 0.90 | 0.028 |  | 0.31 | 0.11 to 0.85 | 0.022 |
| Higher technical school or college |  | 0.28 | 0.10 to 0.76 | 0.013 |  | 0.27 | 0.09 to 0.79 | 0.016 |
| University |  | 0.25 | 0.09 to 0.68 | 0.006 |  | 0.23 | 0.08 to 0.65 | 0.006 |
| **Employment** | *424* |  |  |  |  |  |  |  |
| Employed |  | — | — |  |  | — | — |  |
| Student |  | 0.53 | 0.08 to 2.01 | 0.42 |  | 0.6 | 0.09 to 2.41 | 0.53 |
| Retired |  | 1.41 | 0.76 to 2.54 | 0.26 |  | 1.89 | 0.58 to 6.11 | 0.28 |
| Unemployed or other |  | 2.60 | 1.18 to 5.64 | 0.016 |  | 2.53 | 1.12 to 5.63 | 0.023 |
| **Income** | *405* |  |  |  |  |  |  |  |
| <6'000 CHF |  | — | — |  |  | — | — |  |
| 6'000 - 12'000 CHF |  | 0.91 | 0.54 to 1.53 | 0.72 |  | 1.05 | 0.61 to 1.81 | 0.87 |
| >12'000 CHF |  | 0.71 | 0.39 to 1.25 | 0.24 |  | 0.84 | 0.45 to 1.55 | 0.57 |
| *Legend: OR = Odds Ratio, CI = Confidence Interval, ICU = Intensive Care Unit; ^a^ adjusted for age group, sex, initial hospitalization, and initial symptom severity.* | | | | | | | | |
