# Supplementary material for: Burden of post-COVID-19 syndrome and implications for healthcare service planning: A population-based cohort study
Source: PLoS One. 2021 Jul 12;16(7):e0254523. doi: 10.1371/journal.pone.0254523 (PMC8274847; doi:10.1371/journal.pone.0254523)
Supplement: S6 Table — (DOCX) [file pone.0254523.s006.docx]

**S6 Table. Overlap of participants not having recovered or experiencing fatigue, dyspnea, or depression and healthcare use at six to eight months after diagnosis.**

|  | **Healthcare use related to COVID-19** *^a,b^* | |  |  |
| --- | --- | --- | --- | --- |
| **Variable** | **No**, N=251 | **Yes**, N=170 |  | **Overall**, N=431 |
| **Recovery** |  |  |  |  |
| Recovered to normal health status | 214 (68.8%) | 97 (31.2%) |  | 320 (74.2%) |
| Not recovered to normal health status | 37 (33.6%) | 73 (66.4%) |  | 111 (25.8%) |
| **Fatigue (measured by FAS)** |  |  |  |  |
| No | 121 (64.0%) | 68 (36.0%) |  | 193 (45.3%) |
| Yes | 128 (56.1%) | 100 (43.9%) |  | 233 (54.7%) |
| *Missing* | *2* | *2* |  | *5* |
| **Dyspnea (measured by mMRC scale)** |  |  |  |  |
| mMRC grade 0 | 196 (67.4%) | 95 (32.6%) |  | 299 (75.7%) |
| mMRC grade ≥1 | 36 (37.9%) | 59 (62.1%) |  | 96 (24.3%) |
| *Missing* | *19* | *16* |  | *36* |
| **Depression (measured by DASS-21)** |  |  |  |  |
| No | 203 (65.3%) | 108 (34.7%) |  | 317 (74.1%) |
| Yes | 48 (44.4%) | 60 (55.6%) |  | 111 (25.9%) |
| *Missing* | *0* | *2* |  | *3* |

*Legend: FAS = Fatigue Assessment Scale, mMRC = modified Medical Research Council, DASS-21 = Depression, Anxiety and Stress Score (21 items). ^a^ Data on healthcare use missing from 10 individuals, ^b^ row percentages presented.*
