# Supplementary material for: Burden of post-COVID-19 syndrome and implications for healthcare service planning: A population-based cohort study
Source: PLoS One. 2021 Jul 12;16(7):e0254523. doi: 10.1371/journal.pone.0254523 (PMC8274847; doi:10.1371/journal.pone.0254523)
Supplement: S7 Table — (DOCX) [file pone.0254523.s007.docx]

**S7 Table**. **Results from univariable and multivariable logistic regression models for the outcome of having at least one further healthcare contact (defined as rehospitalization, general practitioner visit or medical hotline call) related to COVID-19 within six to eight months after diagnosis.**

| **Variable** |  | **Univariable** | | |  | **Multivariable** *^a^* | | |
| --- | --- | --- | --- | --- | --- | --- | --- | --- |
|  | **N** | **OR** | **95% CI** | **p-value** |  | **OR** | **95% CI** | **p-value** |
| **Age group (years)** | *421* |  |  |  |  |  |  |  |
| 18-39 |  | — | — |  |  | — | — |  |
| 40-64 |  | 2.24 | 1.44 to 3.50 | <0.001 |  | 1.94 | 1.22 to 3.10 | 0.005 |
| ≥65 |  | 3.18 | 1.73 to 5.92 | <0.001 |  | 2.23 | 1.12 to 4.45 | 0.022 |
| **Sex** | *421* |  |  |  |  |  |  |  |
| Male |  | — | — |  |  | — | — |  |
| Female |  | 1.49 | 1.01 to 2.20 | 0.047 |  | 1.60 | 1.05 to 2.45 | 0.029 |
| **Time since diagnosis (days)** | *421* | 1.00 | 1.00 to 1.01 | 0.74 |  | 1.00 | 0.99 to 1.00 | 0.26 |
| **Initial symptom severity** | *421* |  |  |  |  |  |  |  |
| Asymptomatic |  | — | — |  |  | — | — |  |
| Mild to moderate |  | 1.46 | 0.72 to 3.19 | 0.31 |  | 1.50 | 0.72 to 3.34 | 0.30 |
| Severe to very severe |  | 3.38 | 1.64 to 7.45 | 0.002 |  | 2.59 | 1.21 to 5.87 | 0.018 |
| **Initial hospitalization** | *421* |  |  |  |  |  |  |  |
| No |  | — | — |  |  | — | — |  |
| Yes |  | 4.21 | 2.52 to 7.21 | <0.001 |  | 2.94 | 1.66 to 5.32 | <0.001 |
| **Initial ICU stay** | *421* |  |  |  |  |  |  |  |
| No |  | — | — |  |  | — | — |  |
| Yes |  | 14 | 2.59 to 259 | 0.013 |  | 3.65 | 0.61 to 70.1 | 0.24 |
| **Recovery** | *421* |  |  |  |  |  |  |  |
| Recovered to normal health status |  | — | — |  |  | — | — |  |
| Not recovered to normal health status | | 4 | 2.76 to 6.97 | <0.001 |  | 3.53 | 2.14 to 5.86 | <0.001 |
| **Fatigue** | *417* |  |  |  |  |  |  |  |
| No |  | — | — |  |  | — | — |  |
| Yes |  | 1 | 0.94 to 2.07 | 0.1 |  | 1.61 | 1.04 to 2.50 | 0.032 |
| **Dyspnea** | *417* |  |  |  |  |  |  |  |
| mMRC grade 0 |  | — | — |  |  | — | — |  |
| mMRC grade ≥1 |  | 1 | 0.94 to 2.07 | 0.1 |  | 2.35 | 1.40 to 3.99 | 0.001 |
| **Depression** | *419* |  |  |  |  |  |  |  |
| No |  | — | — |  |  | — | — |  |
| Yes |  | 2 | 1.51 to 3.68 | <0.001 |  | 2.13 | 1.32 to 3.45 | 0.002 |
| **Smoking status** | *418* |  |  |  |  |  |  |  |
| Non-smoker |  | — | — |  |  | — | — |  |
| Ex-smoker |  | 1.23 | 0.79 to 1.91 | 0.36 |  | 1.14 | 0.70 to 1.84 | 0.59 |
| Smoker |  | 0.86 | 0.47 to 1.54 | 0.61 |  | 1.16 | 0.61 to 2.18 | 0.64 |
| **Body mass index** | *413* | 1.01 | 0.98 to 1.05 | 0.48 |  | 0.99 | 0.94 to 1.03 | 0.57 |
| **Comorbidities** | *420* |  |  |  |  |  |  |  |
| No |  | — | — |  |  | — | — |  |
| Yes |  | 1.80 | 1.20 to 2.72 | 0.005 |  | 1.14 | 0.70 to 1.84 | 0.59 |
| *Legend: OR = Odds Ratio, CI = Confidence Interval, ICU = Intensive Care Unit; ^a^ adjusted for age group, sex, initial hospitalization, and initial symptom severity.* | | | | | | | | |
