# Supplementary material for: Burden of post-COVID-19 syndrome and implications for healthcare service planning: A population-based cohort study
Source: PLoS One. 2021 Jul 12;16(7):e0254523. doi: 10.1371/journal.pone.0254523 (PMC8274847; doi:10.1371/journal.pone.0254523)
Supplement: S8 Table — (DOCX) [file pone.0254523.s008.docx]

**S8 Table. Sensitivity analysis of relative health status, fatigue, dyspnea, mental health, and health-related quality of life in study participants at six to eight months after SARS-CoV-2 infection, stratified into time periods of limited and increased testing for SARS-CoV-2, as well as with limited and increased awareness of post-COVID-19 syndrome.**

|  | **Test timing (25 Jun 2020)** | |  | **Awareness (09 Nov 2020)** | |  |  |
| --- | --- | --- | --- | --- | --- | --- | --- |
| **Variable** | **Limited testing**, N=313 | **Increased testing**, N=117 |  | **Lower awareness of post-COVID syndrome**, N=129 | **Increased awareness of post-COVID syndrome**, N=302 |  | **Overall**, N=431 |
| **Recovery** |  |  |  |  |  |  |  |
| Recovered to normal health status | 237 (75.7%) | 82 (70.1%) |  | 102 (79.1%) | 218 (72.2%) |  | 320 (74.2%) |
| Not recovered to normal health status | 76 (24.3%) | 35 (29.9%) |  | 27 (20.9%) | 84 (27.8%) |  | 111 (25.8%) |
| **Self-reported symptoms** |  |  |  |  |  |  |  |
| No new or ongoing symptoms | 235 (75.1%) | 89 (76.1%) |  | 95 (73.6%) | 230 (76.2%) |  | 325 (75.4%) |
| Any new or ongoing symptoms | 78 (24.9%) | 28 (23.9%) |  | 34 (26.4%) | 72 (23.8%) |  | 106 (24.6%) |
| **Recovery and symptoms** |  |  |  |  |  |  |  |
| Recovered and symptom-free | 197 (62.9%) | 67 (57.3%) |  | 83 (64.3%) | 182 (60.3%) |  | 265 (61.5%) |
| Not recovered or experiencing symptoms | 116 (37.1%) | 50 (42.7%) |  | 46 (35.7%) | 120 (39.7%) |  | 166 (38.5%) |
| **Fatigue (measured by FAS)** |  |  |  |  |  |  |  |
| No fatigue | 144 (46.8%) | 49 (41.9%) |  | 69 (54.3%) | 124 (41.5%) |  | 193 (45.3%) |
| Fatigue | 164 (53.2%) | 68 (58.1%) |  | 58 (45.7%) | 175 (58.5%) |  | 233 (54.7%) |
| *Missing* | *5* | *0* |  | *2* | *3* |  | *5* |
| **Dyspnea (measured by mMRC scale)** |  |  |  |  |  |  |  |
| mMRC grade 0 | 212 (74.4%) | 86 (78.9%) |  | 87 (73.1%) | 212 (76.8%) |  | 299 (75.7%) |
| mMRC grade 1 | 59 (20.7%) | 22 (20.2%) |  | 29 (24.4%) | 52 (18.8%) |  | 81 (20.5%) |
| mMRC grade ≥2 | 14 (4.9%) | 1 (0.9%) |  | 3 (2.5%) | 12 (4.3%) |  | 15 (3.8%) |
| *Missing* | *28* | *8* |  | *10* | *26* |  | *36* |
| **Depression (measured by DASS-21)** |  |  |  |  |  |  |  |
| No depression | 233 (75.2%) | 83 (70.9%) |  | 99 (77.3%) | 218 (72.7%) |  | 317 (74.1%) |
| Mild to moderate depression | 59 (19.0%) | 26 (22.2%) |  | 18 (14.1%) | 67 (22.3%) |  | 85 (19.9%) |
| Severe to very severe depression | 18 (5.8%) | 8 (6.8%) |  | 11 (8.6%) | 15 (5.0%) |  | 26 (6.1%) |
| *Missing* | *3* | *0* |  | *1* | *2* |  | *3* |
| **Anxiety (measured by DASS-21)** |  |  |  |  |  |  |  |
| No anxiety | 213 (69.2%) | 77 (65.8%) |  | 93 (73.2%) | 198 (66.2%) |  | 291 (68.3%) |
| Mild to moderate anxiety | 68 (22.1%) | 35 (29.9%) |  | 26 (20.5%) | 77 (25.8%) |  | 103 (24.2%) |
| Severe to very severe anxiety | 27 (8.8%) | 5 (4.3%) |  | 8 (6.3%) | 24 (8.0%) |  | 32 (7.5%) |
| *Missing* | *5* | *0* |  | *2* | *3* |  | *5* |
| **Stress (measured by DASS-21)** |  |  |  |  |  |  |  |
| No stress | 263 (85.7%) | 93 (79.5%) |  | 112 (88.9%) | 245 (81.9%) |  | 357 (84.0%) |
| Mild to moderate stress | 33 (10.7%) | 18 (15.4%) |  | 11 (8.7%) | 40 (13.4%) |  | 51 (12.0%) |
| Severe to very severe stress | 11 (3.6%) | 6 (5.1%) |  | 3 (2.4%) | 14 (4.7%) |  | 17 (4.0%) |
| *Missing* | *6* | *0* |  | *3* | *3* |  | *6* |
| **EQ-5D mobility** |  |  |  |  |  |  |  |
| No mobility problems | 276 (88.7%) | 104 (88.9%) |  | 115 (89.1%) | 266 (88.7%) |  | 381 (88.8%) |
| Mobility problems | 35 (11.3%) | 13 (11.1%) |  | 14 (10.9%) | 34 (11.3%) |  | 48 (11.2%) |
| *Missing* | *2* | *0* |  | *0* | *2* |  | *2* |
| **EQ-5D self care** |  |  |  |  |  |  |  |
| No problems with self-care | 310 (99.4%) | 117 (100.0%) |  | 129 (100.0%) | 299 (99.3%) |  | 428 (99.5%) |
| Problems with self-care | 2 (0.6%) | 0 (0.0%) |  | 0 (0.0%) | 2 (0.7%) |  | 2 (0.5%) |
| *Missing* | *1* | *0* |  | *0* | *1* |  | *1* |
| **EQ-5D usual activities** |  |  |  |  |  |  |  |
| No problems during usual activities | 278 (89.1%) | 106 (90.6%) |  | 117 (90.7%) | 268 (89.0%) |  | 385 (89.5%) |
| Problems during usual activities | 34 (10.9%) | 11 (9.4%) |  | 12 (9.3%) | 33 (11.0%) |  | 45 (10.5%) |
| *Missing* | *1* | *0* |  | *0* | *1* |  | *1* |
| **EQ-5D pain & discomfort** |  |  |  |  |  |  |  |
| No pain or discomfort present | 192 (61.7%) | 84 (72.4%) |  | 84 (65.1%) | 193 (64.5%) |  | 277 (64.7%) |
| Pain or discomfort present | 119 (38.3%) | 32 (27.6%) |  | 45 (34.9%) | 106 (35.5%) |  | 151 (35.3%) |
| *Missing* | *2* | *1* |  | *0* | *3* |  | *3* |
| **EQ-5D anxiety & depression** |  |  |  |  |  |  |  |
| No anxiety or depression present | 222 (71.2%) | 75 (64.1%) |  | 85 (65.9%) | 212 (70.4%) |  | 297 (69.1%) |
| Anxiety or depression present | 90 (28.8%) | 42 (35.9%) |  | 44 (34.1%) | 89 (29.6%) |  | 133 (30.9%) |
| *Missing* | *1* | *0* |  | *0* | *1* |  | *1* |
| **EQ-5D-5L index score** |  |  |  |  |  |  |  |
| Median (IQR) | 0.89 (0.84 to 1.00) | 0.89 (0.85 to 1.00) |  | 0.89 (0.83 to 1.00) | 0.89 (0.85 to 1.00) |  | 0.89 (0.85 to 1.00) |
| Range | 0.07 to 1.00 | 0.41 to 1.00 |  | 0.44 to 1.00 | 0.07 to 1.00 |  | 0.07 to 1.00 |
| *Missing* | *3* | *1* |  | *0* | *4* |  | *4* |
| **EQ VAS** |  |  |  |  |  |  |  |
| Median (IQR) | 85 (77 to 90) | 85 (77 to 90) |  | 85 (77 to 90) | 85 (77 to 90) |  | 85 (77 to 90) |
| Range | 20 to 100 | 40 to 100 |  | 50 to 100 | 20 to 100 |  | 20 to 100 |
| *Missing* | *8* | *2* |  | *5* | *5* |  | *10* |

*Legend: FAS = Fatigue Assessment Scale, mMRC = modified Medical Research Council, DASS-21 = Depression, Anxiety and Stress Score (21 items), EQ = EuroQol, VAS = Visual Analogue Scale, IQR = Interquartile Range.*
