# Supplementary material for: Burden of post-COVID-19 syndrome and implications for healthcare service planning: A population-based cohort study
Source: PLoS One. 2021 Jul 12;16(7):e0254523. doi: 10.1371/journal.pone.0254523 (PMC8274847; doi:10.1371/journal.pone.0254523)
Supplement: S1 Fig — (DOCX) [file pone.0254523.s009.docx]

**S**
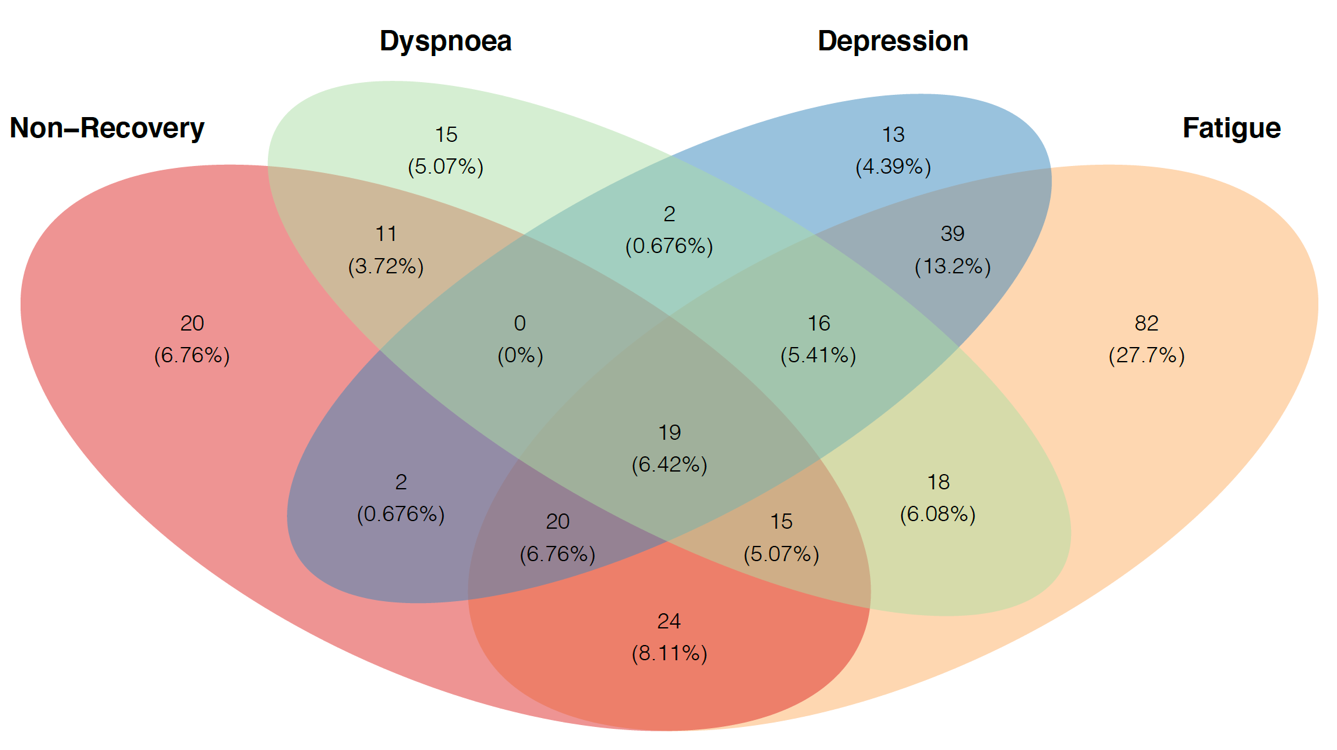
**1 Fig**. **Venn diagram of the overlap of participants that have not fully recovered or are experiencing fatigue, dyspnea or depression at six to eight months after SARS-CoV-2 infection (total N=296).**
