# Supplementary material for: Burden of post-COVID-19 syndrome and implications for healthcare service planning: A population-based cohort study
Source: PLoS One. 2021 Jul 12;16(7):e0254523. doi: 10.1371/journal.pone.0254523 (PMC8274847; doi:10.1371/journal.pone.0254523)
Supplement: Attachment — Submitted filename: ZSAC_PostCovid Syndrome_Response to Reviewers_R2_20210621.docx [file pone.0254523.s012.docx]

|  | **Milo A Puhan, MD, PhD**  Prof. and Chair of Epidemiology  Director EBPI, University of Zurich  President Swiss School of Public Health  Adjunct Professor, Johns Hopkins Bloomberg School of Public Health, Dep. of Epidemiology  Email: [miloalan.puhan@uzh.ch](mailto:miloalan.puhan@uzh.ch) |
| --- | --- |
| **Editorial Board**  PLOS ONE |  |
| Zurich, 21 June 2021 | |

**Point-by-point responses to reviewer comments (PONE-D-21-12057)**

**Comments to the Author**

**Journal Requirements**

Please review your reference list to ensure that it is complete and correct. If you have cited papers that have been retracted, please include the rationale for doing so in the manuscript text, or remove these references and replace them with relevant current references. Any changes to the reference list should be mentioned in the rebuttal letter that accompanies your revised manuscript. If you need to cite a retracted article, indicate the article’s retracted status in the References list and also include a citation and full reference for the retraction notice.

We revised the publication list and updated the references for Sudre et al., 2021 (replaced medrxiv preprint with publication in Nature Medicine), as well as Mandal et al., 2021 and Al-Aly et al., 2021 (updated reference to most recent record). To our best knowledge, none of the cited articles has been retracted.

**Reviewer #1**

After the revision the manuscript „Burden of post-COVID-19 syndrome and implications for healthcare service planning: a population-based cohort study” (PONE-D-21-12057_R1) by Menges and colleagues has improved substantially. My questions were answered satisfactorily for the most part. The remaining gaps are adequately discussed by the authors in the limitations.

Thank you very much for your thorough review of our study and your positive feedback. We are happy that we were able to address your concerns to your satisfaction.

Please correct line 417 “On one side,...” instead of “One one side,…”

We have corrected this mistake.

**Reviewer #2**

All my previous comments were answered and I am satisfied with the responses. I just have the following final comments:

Thank you very much for your insightful comments and your positive feedback. We are happy that we were able to satisfactorily respond to your concerns.

Comment 1: Line 168 – 170 the sentence looks incomplete. I think it is missing the word “bias”. As in “to assess potential selection bias”.

We changed the wording to "to assess potential selection bias.".

Comment 2: Table 1 Kindly put units in brackets for the variable “initial symptom duration” (days). just like you had put for the variable “time since diagnosis (days”.

Thank you for raising this to our attention. We included the units for initial symptom duration (days) and BMI (kg/m^2^) in Table 1, as well as for time from symptom onset to diagnosis (days) in Table S1.

Comment 3: The multivariate logistics regression analysis results in table 2 are ok and well presented. However, there is possible misallocation of the reference group or level for some of the significant covariables. For example, the covariable “gender” for outcome “non-recovery” and “dyspnea”. Also the covariable age group for outcome “fatigue” etc. It is preferable that the level with the lower proportion of the outcome variable should be the reference level for that covariable. However, this does not change the significance of the results and it may only be a matter of preference of reporting.

We agree that the results for regression analyses may be easier to interpret with a different allocation of the reference group for some variables. We changed the reference for sex in all analyses (Fig 2-3; S2-S5 Table, S8 Table), in line with your suggestions and the reporting in the text. Meanwhile, our preference would be to keep the current reporting for the other variables, for the following reasons: First, we would favor to use consistent reference levels for variables with varying direction of associations across analyses for the different outcomes (e.g. for age group, ICU stay). Second, we would prefer using the lowest levels for variables of ordinal nature (e.g. for age group, income, education), and the most frequent level (e.g. "employed") for employment. We investigated your suggestion and feel that our data is more interpretable as it is currently presented. Also, the precision of the respective estimates is negatively affected when changing the reference level (e.g. since the group of students and university-educated individuals is rather small). In our resubmission, we include both our suggested version for the figures and tables, as well as an alternative version (version 2) more in line with your suggestions. We would like to leave it up to the editor to decide which presentation of the data is preferred for publication.

Comment 4: looking at the results in figure 2, isn’t the covariable “presence of comorbidity” also significantly associated with outcome “dyspnea”?

This is correct - we added a statement regarding associations of the presence of comorbidities and chronic respiratory conditions with dyspnea to the manuscript (lines 246-248).
